# Phenotypic plasticity of vascular smooth muscle cells in vascular calcification: Role of mitochondria

**DOI:** 10.3389/fcvm.2022.972836

**Published:** 2022-10-12

**Authors:** Yan Zhong Liu, Zong Xiang Li, Lin Lin Zhang, Dan Wang, Yi Ping Liu

**Affiliations:** Provincial University Key Laboratory of Sport and Health Science, School of Physical Education and Sport Sciences, Fujian Normal University, Fuzhou, China

**Keywords:** vascular calcification, vascular smooth muscle cell (VSMC), mitochondria, phenotypic switch, cardiovascular disease

## Abstract

Vascular calcification (VC) is an important hallmark of cardiovascular disease, the osteo-/chondrocyte phenotype differentiation of vascular smooth muscle cells (VSMCs) is the main cause of vascular calcification. Accumulating evidence shows that mitochondrial dysfunction may ultimately be more detrimental in the VSMCs calcification. Mitochondrial participate in essential cellular functions, including energy production, metabolism, redox homeostasis regulation, intracellular calcium homeostasis, apoptosis, and signal transduction. Mitochondrial dysfunction under pathological conditions results in mitochondrial reactive oxygen species (ROS) generation and metabolic disorders, which further lead to abnormal phenotypic differentiation of VSMCs. In this review, we summarize existing studies targeting mitochondria as a treatment for VC, and focus on VSMCs, highlighting recent progress in determining the roles of mitochondrial processes in regulating the phenotype transition of VSMCs, including mitochondrial biogenesis, mitochondrial dynamics, mitophagy, mitochondrial energy metabolism, and mitochondria/ER interactions. Along these lines, the impact of mitochondrial homeostasis on VC is discussed.

## Introduction

Vascular calcification (VC), the ectopic deposition of calcium (Ca^2+^) apatite within the vascular wall, is prevalent in chronic kidney disease and diabetes mellitus and contributes to subsequent cardiovascular morbidity and mortality (1). Calcification occurs in both the intimal and medial layers of the arteries. Intimal calcification is linked to arterial obstruction and atherosclerotic plaque rupture. In contrast, medial calcification is linked to vessel stiffness, systolic hypertension, and increased pulse wave velocity leading to increased diastolic dysfunction and heart failure (2). Several meta-analyses have shown that aortic and coronary artery calcification predicts overall mortality in high-risk populations and leads to a multifold increase in cardiovascular and cerebrovascular mortality (3–5).

Calcification of the vascular medial layer, initially thought to be a passive process is actually an active, tightly regulated process driven primarily by vascular smooth muscle cells (VSMCs). VSMCs play an indispensable role in the phenotypic transition of chondroblasts and osteoblasts and lead to VC (6). The molecular mechanism underlying phenotypic transition of VSMCs is complex, mainly including elevated intracellular Ca^2+^ concentration (7), oxidative stress (8), and metabolic reprogramming (9). However, the regulatory mechanisms among these factors remain largely elusive. Notably, growing evidence indicates a vital role of mitochondrial in phenotypic transition of VSMCs (10–13). Mitochondrial constantly integrate signals from environment and respond accordingly to match vascular function to metabolic requirements of the organ tissue, and play a key role in energy production, redox homeostasis, Ca^2+^ homeostasis, and signal transduction, while mitochondrial dysfunction contributes to altered metabolism, oxidative stress, and increased apoptosis in VSMCs (14). Regardless of apoptosis or necrosis, cellular components such as Ca^2+^ and DNA are released after cell death, resulting in the deposition of Ca^2+^ and phosphorus, which may lead to VC (2). Following recent new insight in the mitochondrial function of VSMCs in VC, we believe it is time to focus on the complex role of mitochondrial homeostasis in phenotypic transition of VSMCs. In this review, we discuss the distinct phenotypes that VSMCs acquire in VC, and highlight the role of mitochondria in maintaining VSMCs function.

## Phenotypic switching of vascular smooth muscle cells in vascular calcification

**FIGURE 1:**
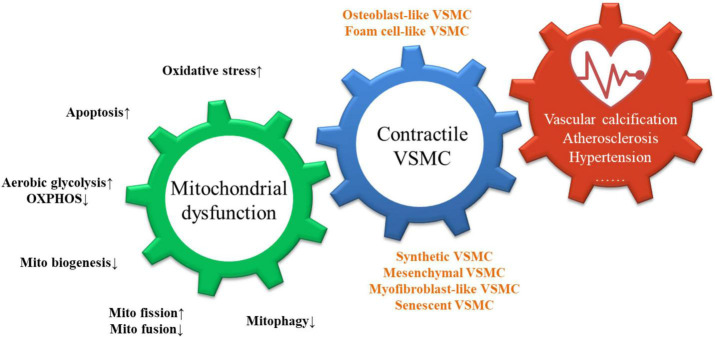
Consequences of mitochondrial dysfunction in VSMCs.

Vascular smooth muscle cells are the predominant cell type of blood vessels, alternating with elastic fibrous layers to form the medial layer of vascular, they can produce contraction and relaxation responses through the interaction of myosin and actin, and regulate blood pressure and blood flow distribution in various parts of the body. Unlike terminally differentiated cells such as cardiomyocytes and skeletal muscle cells, even mature VSMCs exhibit a high degree of phenotypic plasticity. VSMCs showed no significant proliferation, migration, and secretion of extracellular matrix under healthy physiological conditions, known as differentiated and contractile phenotype. In response to various pathological stimuli and biochemical regulations such as mechanical forces, vasoactive agents, changes in pyrophosphate levels, inflammatory mediators, and oxidative stress, VSMCs can re-enter the cell cycle and switch from a differentiated and contractile phenotype to dedifferentiated and synthetic phenotype, characterized by a reduction in marker of a range of cytoskeletal proteins such as smooth muscle myosin heavy chain (SM-MHC), α-SMA, and smooth muscle 22α (SM-22α). Previously, a binary model of VSMCs phenotype switching was established whereby transforming growth factor-β (TGF-β) stimulation promoted a quiescent “contractile” VSMCs phenotype, whereas platelet-derived growth factor-BB (PDGF-BB) or Angiotensin I (Ang II) stimulation triggered to the adoption of a pro-migratory, hyper-proliferative “synthetic” phenotype, which contributed to vascular diseases (15). However, the features of the contractile phenotype of VSMCs were evident, while the non-contractile phenotype displayed multiple characteristics. More recently it has become clear that under specific pathological conditions, VSMCs can maintain a spectrum of phenotypes and can express feature of osteoblasts, chondrocytes (6), macrophage-like (16, 17), foam cells (18), adipocyte-like (19), fibroblast-like (20), and pluripotent vascular stem cells (21). This plasticity of the VSMCs phenotype is associated with various vascular diseases including VC, atherosclerosis, and hypertension (Figure 1). The phenotype of VSMCs in VC was found to switch toward an osteo/chondrocyte phenotype, which is characterized by decrease in contractile protein expression and upregulation of mineralization-related markers such as runt-related transcription factor 2 (RUNX2), osterix (OSX), osteopontin (OPN), osteocalcin (OX), alkaline phosphatase (ALP), collagen type II, and type χ (22). Although the phenotypic transformation of VSMCs is thought to predate the development of vascular diseases including VC, the molecular mechanisms leading to the loss of the contractile phenotype and the upregulation of markers of the osteo/chondrocyte phenotype are at present not fully understood. There have been several recent studies assessing the beneficial role of mitochondria in maintaining redox, Ca^2+^ homeostasis and metabolic regulation (Table 1). Thus, targeting mitochondria to regulate VSMCs phenotypic transition provides a potential therapeutic direction for alleviating VC.

**TABLE 1:** Summary of targeted mitochondria in the treatment of vascular calcification.

Therapeutic interventions	Models	Alterations in mitochondria	Target molecules	Main effects on VSMCs	References
Melatonin	VSMCs subjected to β-GP	↓Mitochondrial fission ↓mitochondrial superoxide ↓mPTP, ↓apoptosis ↑ATP synthesis, ΔΨm	AMPK, Drp1	↓RUNX2 ↓ALP	(23)
Quercetin	Rat model of adenine-induced; VSMCs subjected to Pi	↓Mitochondrial ROS levels ↓cytochrome c release ↓apoptosis ↓mitochondrial fission ↑ATP synthesis, ΔΨm Improve cristae shape	Drp1, p-Drp1	Decrease calcium deposition	(24)
Irisin	Mice model of adenine-induced; VSMCs subjected to β-GP	↓Fission (Drp1 expression) ↓apoptosis, MDA, ROS levels ↑SOD ↑ATP synthesis, ΔΨm ↑fusion (Mfn2 expression) Improve mitochondrial morphology	AMPK, Drp1, Mfn2	↓RUNX2 ↓BMP-2 ↑α-SMA ↑SM-22α	(25)
Mitoquinone	Rat model of adenine-induced; VSMCs subjected to Pi	↓MDA, ROS levels ↓apoptosis ↑SOD	Nrf2/Keap1, Bcl-2, Bax	Decrease calcium deposition	(26)
α-lipoic acid	VSMCs subjected to Pi	↓Cytochrome c release ↓apoptosis ↓superoxide ↑ATP synthesis, ΔΨm Improve cristae shape	Gas6/Axl/Akt	Decrease calcium deposition	(27)
Dextromethorphan	Rat model of adenine-induced; VSMCs subjected to β-GP	↓ROS levels ↑ATP synthesis, ΔΨm	NADPH oxidase	↓RUNX2 Decrease calcium deposition	(28)
Sodium citrate*; Diethyl citrate	VSMCs subjected to nano-HAp	↓ROS levels ↓apoptosis ↓[Ca^2+^]i ↓lysosomal damage ↑ΔΨm	LDH, Ca^2+^	-	(29)
Metformin	VSMCs subjected to β-GP	↓Apoptosis ↓MDA, ROS levels ↑SOD, ↑mitophagy ↑ATP synthesis, ΔΨm ↑mitochondrial biogenesis (density, mass, mtDNA)	AMPK, PGC-1α, Nrf1, Tfam, PDK4, LC3-↑, p62, Atg5	↓RUNX2, BMP-2 ↓osteocalcin ↓ALP ↑SM-22α	(30)
HSP72	Human VSMCs subjected to β-GP	↓Apoptosis ↓cytochrome c release ↑ΔΨm	P53, Bax, Bcl-2	Inhibited the development of VC	(31)
Resveratrol	VSMCs subjects to DMEM, β-GP, L-ascorbic acid, dexamethasone	↓ROS levels ↓apoptosis ↓[Ca^2+^]i ameliorate mitochondrial dysfunction	Sirt1, Nrf2, FGF-23	Decrease calcium deposition ↓RUNX2, OPN ↓HO-1	(32)
Antioxidant (*N*-acetyl-L-cysteine)	VSMCs subjects to Hydroxyapatite nanoparticles	↓ROS levels ↓NLRP3, caspase1, GSDMD-N, IL-1β, IL-18, TNF-α ↓mPTP ↓apoptosis	NLRP3/caspase-1/gasdermin D	↓RUNX2 ↓ALP ↓COL-1α	(33)
Calpain inhibitor I; Mito-TEMPO	Rats with hypercholesterolemia; VSMCs treated with ox-LDL	↓Superoxide anion ↑ATP synthase activity, ATP5D ↑PPi	Calpain-1	Decrease calcium deposition ↓ALP	(34)

VC, vascular calcification; VSMCs, vascular smooth muscle cells; ATP, adenosine triphosphate; AMPK, AMP-activated protein kinase; ΔΨm, mitochondrial membrane potential; β-GP, β-glycerophosphate; mPTP, mitochondrial permeability transition pore; Pi, phosphate; mitochondrial permeability transition pore; Drp1, dynamin-related peptide 1; RUNX2, runt-related transcription factor 2; ALP, alkaline phosphatase; MDA, malondialdehyde; ROS, reactive oxygen species; Mfn2, Mitofusin 2; SOD, superoxide dismutase; BMP-2, bone morphogenetic protein-2; α-SMA, α-smooth muscle actin; SM-22α, smooth muscle protein 22-α; Nrf2, nuclear factor E2-related factor 2; Keap1, kelch-like ECH-Associating protein 1; Bcl-2, B-cell lymphoma-2; Bax, Bcl2-Associated X; Gas6, growth arrest-specific protein 6; Axl, Gas6 cognate receptor; Akt, protein kinase B; NADPH, nicotinamide adenine dinucleotide phosphate; ASMCs, aortic smooth muscle cells; Nano-Hap, nanosized hydroxyapatite; *the treatment effect is greater than the other; LDH, lactate dehydrogenase; [Ca^2+^]i, intracellular Ca^2+^; mtDNA, mitochondrial DNA; PGC-1α, peroxisome proliferator activator receptor gamma coactivator 1 α; Nrf1, nuclear respiratory factor 1; Tfam, mitochondrial transcription factor A; PDK4, pyruvate dehydrogenase kinase4; Atg5, autophagy protein 5; DMEM, Dulbecco’s modified Eagle medium; OPN, osteopontin; FGF-23, fibroblast growth factor 23; HO-1, heme oxygenase-1; COL-1α, type ↑ collagen; PPi, pyrophosphate.

As shown, mitochondrial dysfunction is manifested by an increase in oxidative stress, apoptosis, aerobic glycolysis, mitochondrial fission, and a decrease in OXPHOS, mitochondrial biosynthesis, mitochondrial fusion, and mitophagy. These triggers interact and act synergistically to promote the loss of the contractile phenotype of VSMCs and the transition to osteoblast-like, foam-cell-like phenotypes, leading to the development of vascular diseases including vascular calcification, atherosclerosis, and hypertension.

## Mitochondrial homeostasis and phentypic transition of vascular smooth muscle cells

### Mitochondrial biogenesis

Mitochondria perform diverse yet interconnected functions, producing ATP and many biosynthetic intermediates to meet the needs of the cell’s metabolic capacity and also contribute to the cellular stress response. Under appropriate conditions, in response to oxidative stress, mitochondrial dysfunction, and an increase in the energy requirements of the cells, cells can stimulate functional mitochondrial proliferation *via* mitochondrial biogenesis to re-establish redox, energy metabolism, and counter-inflammatory milieu homeostasis to rejuvenate the number and size of mitochondria and prevent cell death (35). Thus, mitochondrial biogenesis is defined as the process *via* which cells increase their mitochondrial mass (36). If this process is blocked, not only the cell energy regulation will be out of control, but also due to mitochondrial dysfunction and activation of apoptosis, mitochondria will produce excessive reactive oxygen species, resulting in oxidative stress damage and calcium concentration imbalance, which in turn induces the osteogenic differentiation and calcification of VSMCs (37). Correct mitochondrial biogenesis is very complex and requires the coordinated synthesis of mitochondrial DNA (mtDNA) encoded protein, import of proteins encoded by the nuclear genome and synthesized on cytosolic ribosomes, assembly of the dual genetic origin derived proteins, as well as mtDNA replication (38, 39).

Mitochondrial biogenesis is regulated mainly at the level of transcription. The peroxisome proliferator-activated receptor-γ coactivator (PGC)-1α, the most well-known, is thought to act as a master regulator of mitochondrial biogenesis that controls the optimal mitochondrial content by interacting with various transcription factors, such as peroxisome proliferator-activated receptors (PPARs), mitochondrial transcription factor A (TFAM) (40), estrogen-related receptor (ERR) (41), nuclear respiratory factors (Nrfs), and uncoupling protein 2 (UCP2) (42). PGC-1α cooperatively activates the transcription of Nrf1/2, which in turn regulate the transcription of TFAM. TFAM translocates to the mitochondrial matrix and stimulates mitochondrial DNA replication and mitochondrial gene expression. β-Glycerophosphate (β-GP) a widely used inducer of VC leads to phenotypic transition of VSMCs. Recent evidence shows that in a model of β-GP-induced calcification in VSMCs, a significant reduction in mitochondrial density is displayed, accompanied by mtDNA content, cellular ATP concentration, and expression of mitochondrial biogenesis-related genes such as PGC-1α, Nrf1, and Tfam decreased, suggesting impairment of mitochondrial biogenesis (30). AMP-activated protein kinase (AMPK) and sirtuin1 (Sirt1) are two energy sensors that directly affect PGC-1α activity through phosphorylation and deacetylation, respectively, acting as a crucial link in the maintenance of mitochondrial homeostasis. Pharmacological agents such as metformin or resveratrol can restore mitochondrial biogenesis-related gene expression by activating the AMPK/PGC-1α, Sirt1/Nrf2 signaling pathway, thereby counteracting the osteogenic phenotype transformation of VSMCs. Interestingly, mitophagy is required for metformin-induced mitochondrial biogenesis in VSMCs (30), this suggests that mitophagy and mitochondrial biogenesis are coordinated and that proper mitochondrial quality control is critical for the maintenance of cell function. Meanwhile, decreased expression of PGC-1α and Nrf1/2 in VSMCs proliferation and migration is a common feature of vascular diseases such as atherosclerosis and vascular calcification (32, 43). As the key regulator of mitochondrial biogenesis, activation of Sirt1 can alleviate VSMCs mineralization by upregulating the expression of the calcification inhibitors OPN and osteoprotegerin (OPG), Sirt1 directly acts on the RUNX2 level in hyperglycemic conditions by deacetylation of the RUNX2 promoter (44). During mitochondrial biogenesis, Sirt1 participates inside the nucleus in the induction of PGC-1α that is pivotal to orchestrate the activation of a broad set of transcription factors and nuclear hormone receptors enhancing the expression of the nucleus-encoded mitochondrial genes. In addition to regulating transcription of nucleus-encoded mitochondrial genes, Sirt1 also present as free protein in mitochondria with PGC-1α, and interacts with Tfam. In mitochondria, Sirt1 and PGC-1α form several multiprotein complexs such as PGC-1α/SIRT1, TFAM/PGC-1α, and TFAM/SIRT1. Furthermore, both PGC-1α and Sirt1 reasonably participate with Tfam in the formation of higher molecular weight multiprotein complex, which could correspond to macro-complexes involved in mtDNA transactions (45).

### Mitochondrial dynamics

Mitochondria as highly dynamic organelles can be known as isolated organelles or joined to form larger dynamic networks, they can also be distributed unevenly in the cytosol to meet local energy demands of the cell, this changeable and adaptable nature of mitochondria involving their morphology and subcellular distributions is collectively called mitochondrial dynamics (46). Mitochondrial fusion is the union of two mitochondria resulting in one mitochondrion, it requires three large GTP-hydrolyzing enzymes of the dynamin superfamily: Mitofusion1 (Mfn1), Mfn2, Optic atrophy 1 (Opa1). Mfn1, Mfn2 are located on the mitochondrial outer membrane and are required for outer membrane fusion, and Opa1 is associated with the inner membrane, Cells lacking Opa1 were found to mitochondrial outer membrane fusion, but such events did not progress to inner membrane fusion (47, 48). Mitochondrial fission is the division of one mitochondrion into two smaller mitochondria. The critical mediator of mitochondrial fission is Dynamin-related protein 1 (Drp1), a large GTPase, that is recruited to the mitochondrial outer membrane *via* a collection of receptor proteins, including Mitochondrial fission factor (Mff), Mitochondrial dynamics protein of 49 kDa and 51 kDa (MiD49, MiD50), and Fission protein 1 (Fis1). Once on mitochondria, Drp1 assembles around the tubule and constricts it in a GTP-dependent manner to mediate scission (49). Mitochondrial fission was reported to contribute to mitochondrial apoptosis and was also found to be a prerequisite for mitophagy, while mitochondrial fusion is associated with increased mitochondrial metabolism (50).

The nature of the mitochondrial dynamic network depends on the proper balance between mitochondrial fusion and fission, which are important for biological processes such as maintenance of mitochondrial function, metabolic control, apoptosis, and senescence (51, 52). Fragmented mitochondria clustered in VSMCs often occurs in most vascular diseases, increased mitochondrial fission could result in increased fragmented mitochondria that contribute to the apoptosis-resistant, hyper-proliferative mitochondrial phenotype seen in rat model of spontaneous hypertension (53), pulmonary arterial hypertension (PAH) (54), and atherosclerosis (AS) (55). It is characterized by up-regulated expression of fission proteins (Drp1, Fis1) and down-regulated expression of fusion proteins (Mfn2, Opa1). Drp1 dephosphorylated at serine 637 and phosphorylation increased at serine 616 in PDGF- and Ang II-stimulated VSMCs, and promoted mitochondrial binding of Drp1 to activate mitochondrial fission (56, 57). The mechanism may be that PDGF induces Ca^2+^ release in the endoplasmic reticulum (ER) and triggers extracellular Ca^2+^ influx, and increased intracellular Ca^2+^ ([Ca^2+^]_*i*_) concentration can promote mitochondrial fission through extracellular regulated protein kinase (ERK1/2)-mediated phosphorylation of Drp1 in the cytoplasm (58, 59). In contrast, upregulation of Mfn2 in hyper-proliferative VSMCs exhibits highly anti-proliferative effects on VSMCs *in vivo* and *in vitro* (60), Mfn2 can inactivate the ERK1/2 cascade by blocking the Ras downstream pathway and ultimately arresting the cell cycle in G0/G1 phases (61). However, rat VSMCs treated with H_2_O_2_ induced upregulation of Mfn2 and caspase-3, and caspase-9, showing mitochondria-mediated apoptosis, suggesting that Mfn2 regulates VSMC homeostasis and viability (62). Robert et al. found that in isolated resistance arteries from Opa1^±^ mice, Opa1 haploinsufficiency leads to high mitochondrial fission, mitochondrial cristae disturbance and significantly increased ROS production in VSMC, and upregulation of Mfn1 but not of Mfn2 to compensate for the defect in mitochondrial fusion control caused by Opa1 deficiency (63). In addition, excessive mitochondrial fission exacerbates oxidative stress and disordered energy supply responsible for VSMCs dedifferentiation, directly playing a role in VC. It has been reported that Drp1 expression was significantly increased in human arterial smooth muscle cells compared to non-calcified regions, accompanied by more fragmented mitochondria (64). In a inorganic phosphate (Pi)-induced VSMCs calcification model, increased expression and phosphorylation of Drp1 leads to mitochondrial fragmentation, ROS overproduction, and mitochondrial cristae disorder-mediated apoptosis directly promotes VC (24). Oxidative stress-induced VSMCs matrix mineralization, mitochondrial dysfunction, and ALP activity were attenuated by Drp1 inhibitor.

Mdivi-1, the Drp1 selective inhibitor, is widely used as a pharmacological intervention to inhibit mitochondrial fission process. The treatment of Mdivi-1 can significantly reversed Drp1-induced mitochondrial fission and ROS generation both *in vivo* and *in vitro* (57). In diabetic vascular injury, the imbalance of redox homeostasis leads to the accumulation of ROS and the increase of NADPH oxidase activity, resulting in oxidative stress, which directly leads to the proliferation of VSMCs and further leads to excessive mitochondrial fission. Mdivi-1 can inhibit the expression of Drp1 and the synthesis of ROS in VSMCs under high-glucose conditions, and up-regulate the expression of Mfn2, inhibiting the proliferation of VSMCs (65). Mdivi-1 also effectively reduce the characteristics of osteogenic phenotype transformation such as RUNX2 and type I collagen secretion in the calcification model of VSMCs (64). However, in different VSMCs lesion models, the intervention scheme of Mdivi-1 concentration on mitochondria has not been unified (57, 64), too high Mdivi-1 concentration (50–100 μM) can inhibit the activity of mitochondrial complex I *in vitro* (66), which may be a point that needs to be paid attention to in future research. Recent evidence suggests that the effects of melatonin in β-GP-treated VSMCs were similar to those of Mdivi1. Melatonin reduces VSMC calcium deposition and ALP activity through the AMPK/Drp1 pathway, and mitochondrial fragmentation and superoxide levels are improved, with a concomitant reduction in apoptosis (23). Furthermore, the myokine irisin and antioxidant Quercetin also alleviate VC progression by inhibiting mitochondrial fission through the Drp1 pathway (24, 25). The plant extract salidroside can down-regulate Drp1 and up-regulate Mfn2 in a dose-dependent manner to inhibit mitochondrial fission and restore mitochondrial network homeostasis, reduce ROS levels, and reverse high glucose-induced VSMCs proliferation (67).

In sum, these all results suggest that reducing mitochondrial fission can limit phenotypic transition of VSMCs in VC and rise the possibility that properly manipulating mitochondrial dynamic may be protective in vascular diseases.

### Mitophagy

The impaired or superfluous mitochondria are degraded by a selective form of autophagy, known as mitophagy. As a common component of mitochondrial homeostasis control processes, mitochondrial fission selectively separates aged or damaged mitochondria from the normal mitochondrial pool, which is then targeted by the mitophagy system and transported to lysosomes for degradation and Recycling. Besides general autophagy, mitophagy also plays a role in VC. Recent studies have shown that mitophagy plays a protective role against VC by reversing mitochondrial dysfunction, oxidative stress, and apoptosis (68). Defective mitophagy promotes VSMC apoptosis, and restoration of mitophagy flux can prevent cell death (69).

In mammalian cells, two independent pathways mediate mitophagy. One pathway is mitophagy receptor-mediated and the other is ubiquitin-dependent-mediated (70). Two proteins on the outer membrane of mammalian cells are functionally similar to yeast Atg32 and are suggested to be the two major receptors that mediate mitophagy. BCL2 interacting protein 3 like (BNIP3L, or was called NIX) was originally identified as a mitophagy receptor required for autophagic clearance during erythrocyte maturation. It was recently found that NIX carries a potential LC3-interacting region (LIR) that induces specific mitophagy by binding to the autophagy mediator LC3 (71). The BCL2 interacting protein 3 (BNIP3), a homolog of NIX, was found to mediate lactate-induced osteogenic phenotype transition and calcium deposition in VSMCs. Lactic acid inhibits BNIP3 expression and blocks the binding of LC3-II and BNIP3, reduces mitophagy flux, and causes the backlog of damaged mitochondria, oxidative stress, and mitochondrial permeability transition pore (mPTP)-mediated apoptosis. Overexpression of BNIP3 attenuates high levels of oxidative stress and VC, to some extent (68). FUNDC1 is another mitophagy receptor localized to the outer mitochondrial membrane. FUNDC1 was reported to be involved in Ang-II-induced VSMCV proliferation and migration, which was further exacerbated when FUNDC1 expression was inhibited (72).

Ubiquitin-dependent mitophagy mediated by PTEN-induced putative kinase protein1 (PINK1) and Parkin is one of the most-studied mitophagy mechanisms in mammalian cells. Under normal conditions, PINK1 enters the mitochondria *via* translocases at the outer and inner mitochondrial membranes and is cleaved by mitochondrial matrix enzymes and proteases when crossing the inner mitochondrial membrane, causing it to retrograde into the cytosol and be rapidly degraded by the proteasome (73). However, When mitochondria are damaged, a decrease in mitochondrial membrane potential (MMP) and mitochondrial depolarization inhibit PINK1 degradation, which leads to the accumulation of PINK1 on the impaired mitochondria outer membrane, phosphorylating ubiquitin attached to mitochondrial outer membrane proteins and recruiting Parkin from the cytosol to the mitochondria, inducing the occurrence of mitophagy (50). Notably, PINK1 is expressed at extremely low levels during normal mitochondrial function, and endogenous PINK1 can only be detected at the mitochondrial outer membrane under specific mitochondrial stress conditions. Thus, PINK1 acts as a mitochondrial damage sensor by accumulating on the surface of mitochondria when there is impaired import (74). The study by He et al. showed that PINK1/Parkin expression was significantly up-regulated in apelin-13-induced human aortic VSMCs proliferation *in vitro* and atherosclerotic lesions of ApoE-/- mice *in vivo* (55). And these results were confirmed by another study (75). These evidences suggest that the PINK1/Parkin regulatory axis plays an important role in the VSMCs hyper-proliferative phenotype. In addition, deficiency of PINK1/Parkin resulted in increased caspase activity and the number of apoptotic cells following oxidized-LDL (ox-LDL) treated human VSMCs, after up-regulation of PINK1, mitophagy flux was increased, and the number of apoptotic cells induced by ox-LDL was significantly reduced (76). Interestingly, no enhancement of mitophagy flux was found upon overexpression of Parkin alone, likely indicating that Parkin functions in two distinct mitochondrial pathways to promote cell survival. One pathway requires PINK1 to initiate mitophagy, and the other pathway is Parkin-dependent ubiquitination and proteasomal degradation of pro-apoptotic Bax (76). Besides, the regulation of general autophagy by Sirt1 is well-known, and there is evidence that Sirt1 plays a protective role in cardiac ischemia-reperfusion and bone metabolism disorders through PINK1/Parkin-mediated mitophagy (77, 78). In VC, the decrease of Sirt1 is an important mechanism causing the osteogenic phenotype transition of VSMC (44), but there is currently no direct evidence that Sirt1 regulates VSMC mitophagy, thereby alleviating VC. Metformin can affect mitochondrial biogenesis by increasing mitophagy, thereby protecting against β-GP-induced osteogenic phenotype transformation of VSMCs. All together, mitophagy acted as a mitochondrial homeostasis regulatory mechanism to protect VSMCs from apoptosis induced by factors such as oxidative and metabolic stress, thereby affecting VC.

**FIGURE 2:**
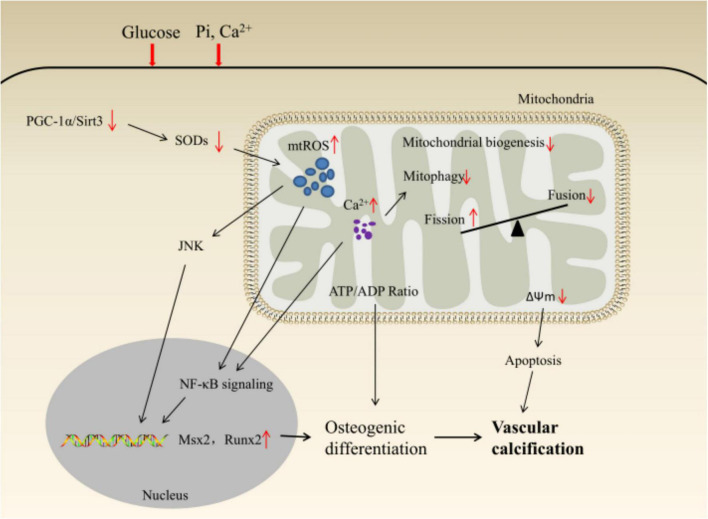
Schematic illustration of mitochondrial homeostasis and VSMCs phenotypic transition.

Under harmful environmental stimuli, the excessive production of mtROS induces the transcription of osteogenic genes (such as Msx2, Runx2) and osteogenic differentiation through JNK or NF-κB pathways; The decrease in ATP/ADP ratio and membrane potential caused by the decrease in mitochondrial quality control activates the caspase-9/3 cascade by promoting the release of cytochrome c, which ultimately leads to VC (Figure 2).

## Consequences of mitochondrial dysfunction in vascular calcification

### Reactive oxygen species generation

Evidence has shown that oxidative stress markers increased with renal function deterioration from the early stages of CKD in children and adults, accompanied by vascular dysfunction (79, 80). Persistent oxidative stress and ROS-induced signaling are key features of cardiovascular disease and are major drivers of VSMCs osteogenic phenotype transformation and VC (81, 82). Mechanistically, H_2_O_2_ could induce VSMC osteogenic differentiation by activating the PI3K/AKT/Runx2 signaling axis and inhibiting the expression of VSMC contractile phenotype markers *in vitro*, and up-regulated the expression of ALP (83, 84). *In vivo* and *in vitro* studies have shown that the increase of MMP leads to ROS production and increases IKKβ phosphorylation and IκBα degradation in a time-dependent manner, thereby inducing the phenotypic transition of VSMC to osteoblast-like phenotype by activating NF-κB and/or Elk-1/serum response factor (SRF) signaling pathways (85). In addition, NF-kB can inhibit the expression of calcification inhibitor ankylosis protein homolog (ANKH) through RNA-destabilizing factor tristetraprolin (TTP)-dependent, and enhance the activity of ALP, then reducing the secretion of PPi and aggravating VC (86). Oxidative stress-dependent ER stress can activate the PERK/eIF2a-ATF4-CHOP signaling pathway to induce the phenotype switch of VSMCs (87), and inhibition of ER stress can restore the contractile phenotype of calcified VSMCs (88). As by-products of mitochondrial oxidative phosphorylation (OXPHOS), mitochondria are the main source of intracellular ROS production (89). Under physiological conditions, the balance between ROS generation and ROS scavenging is highly controlled. Depending on the context, regulated ROS could trigger a variety of cellular responses, including signaling pathways involved in cell survival, initiation coordinated activation of mitochondrial fission and mitophagy to clear abnormal mitochondria (90). The imbalance between ROS production and antioxidant defense mechanisms is responsible for oxidative stress. In the diabetic state, both factors coexist, with reduced endogenous antioxidant defense efficiency and increased reactive oxygen species production both key mechanisms leading to the development of diabetic vascular complications (91). Advanced glycation end products (AGEs) interact with their major receptor RAGE to enhance intracellular oxidative stress levels through NADPH oxidase (NOXs) and mitochondrial pathways (92), inducing VSMCs osteogenic genes expression. The interaction between mitochondria and NOX-derived O_2_^–^ constitutes a feed-forward cycle in which NOX2 increases mtROS production through reverse electron transfer. The increased production of mtROS further activates cytoplasmic NOXs, enhancing the production of cellular O_2_^–^, creating a vicious circle (93). When overexpressing mitochondrial NOX4, mice VSMCs showed decreased expression levels of TFAM and SOD2, indicating mitochondrial biogenesis and antioxidant dysfunction, which may increase ROS levels in a feed-forward manner and induce VSMC apoptosis, Aging, and aortic calcification (94). AGEs-induced upregulation of OPN, OCN, and Runx2 was inhibited by silencing NOX4, and osteogenesis and calcium deposition in VSMCs were reversed (95).

ROS-targeted antioxidant therapy proved to be a viable treatment in an *in vivo* model of VC (96). Evidence suggests that the NADPH oxidase inhibitor dextromethorphan significantly inhibits the increase in ROS production and improves mitochondrial activity, thereby reducing aortic calcification in rats with chronic kidney disease (28). The mitochondria-targeted antioxidant mitoquinone attenuates vascular calcification by inhibiting oxidative stress and apoptosis in VSMCs through the Keap1/Nrf2 pathway (26). Mitochondrial antioxidants (mito-TEMPO) and calpain-1 inhibitors significantly inhibited superoxide anion production in aortic mitochondria, and corrected PPi metabolic disorder, while reducing ALP expression (34). The natural antioxidant α-lipoic acid inhibits VSMC apoptosis *in vitro* and *in vivo*, protects mitochondrial function through antioxidant capacity and restores the Gas6/Axl/Akt survival pathway (27). In addition, mitochondrial uncoupling proteins (U) are considered to be important antioxidants that reduce oxidative stress and oxidative damage by maintaining ROS homeostasis. Zhou et al. showed that activation of peroxisome proliferator-activated receptorγ (PPARγ) could inhibit ROS-induced VSMC proliferation and migration by upregulating UCP2 expression (97). Antioxidants (MnTMPyP, SOD1/2) and mitochondrial respiratory chain inhibitors (rotenone, CCCP, UCP2) can attenuate Pi-induced ROS generation and calcium deposition (85). Pi is an important substrate for ATP synthesis in the process of OXPHOS and participates in various biological functions such as signal transduction and energy metabolism. Apurinic/pyrimidine endonuclease 1/redox factor-1 (APE1/Ref-1) is a multifunctional protein that plays a pleiotropic role in the control of cellular responses to oxidative stress, Gene silencing of APE1/Ref-1 expression reduces basal DNA repair functions and redox activity and is involved in Pi-induced VC, APE1/Ref-1 overexpression significantly inhibits Pi-induced mtROS production, apoptosis, and calcification of VSMCs *in vitro* and *in vivo* (98). It should be noted that although anti-oxidants can all reduce superoxide levels, there are differences in their effect on vascular calcification, which may suggest that scavenging free radicals *in vivo* alone is not enough and that its production may need to be inhibited at the source (96).

### Ca^2+^ handling

Metabolic disturbance of Ca^2+^ is a common phenomenon in diabetes and CKD (99, 100). The increase in cyclic Ca^2+^ is a risk factor for vascular disease and mortality. Serum Ca^2+^ has increased by approximately 0.1 mmol/L, the risk ratio of death is 1.13 (101). The source, amplitude, frequency, and duration of Ca^2+^ signaling are important determinants of VSMCs phenotypic transition (102, 103). [Ca^2+^]_*i*_ is mediated by extracellular Ca^2+^ influx and intracellular Ca^2+^ release. High Pi induces persistent depolarization of the plasma membrane through PiT-1 and -2, the latter activates voltage-gated Ca^2+^ channels (VGCC) leading to Ca^2+^ influx. Mitochondria are the important hub for intracellular Ca^2+^ signaling networks. Mitochondrial Ca^2+^ uptake plays a key role in the normal physiological functions of cells, including ATP production, ROS generation, autophagy, rectification of intracytoplasmic calcium signaling, and regulation of cell death (104, 105).

Ca^2+^ uptake by mitochondria is a process that relies on a gated mitochondrial Ca^2+^ uniporter (MCU) complex. MCU is a low-affinity, high-selectivity Ca^2+^ channel that localizes to the inner mitochondrial membrane (IMM) and is responsible for mitochondrial Ca^2+^ uptake (106). Recent evidence suggests that MCU-dependent mitochondrial Ca^2+^ overload leads to overproduction of mtROS (37), which promotes Drp1-dependent mitochondrial fission and mitophagy, thereby inducing VSMC proliferation, and inhibition of MCU-dependent mitochondrial Ca^2+^ uptake might ameliorate or reverse abnormal VSMC proliferation (58, 107). In addition, mitochondrial Ca^2+^ activates Ca^2+^-sensitive dehydrogenases in the tricarboxylic acid cycle such as pyruvate dehydrogenase, glycerol phosphate dehydrogenase, isocitrate dehydrogenase, and α-ketoglutarate dehydrogenase (108) and downregulating NAD^+^/NADH ratio and NAD^+^-dependent Sirt3 activity further inhibits SOD2 activity, mediating activation of the JNK pathway to increase mtROS production (106), the increased mtROS leads to the opening of mitochondrial permeability transition pore (mPTP), which promotes the release of apoptotic factors such as cytochrome c into the cytoplasm through ruptured mitochondria, and finally triggers cell death (104, 109). Activating the AMPK/PGC-1α/sirtuin-3 (Sirt3) pathway can inhibit MCU-induced mitochondrial Ca^2+^ overload, alleviate mtROS accumulation, stabilize mitochondrial membranes, and promote mitochondrial fusion to improve mitochondrial quality control (110).

In addition, due to the limitation of MCU, mitochondria can only take up Ca^2+^ at high concentrations of Ca^2+^, so Ca^2+^ is most likely to be directly transferred from ER to mitochondria through mitochondria-associated ER membranes (MAMs) and control ATP production and apoptosis (111). ER is the main intracellular organelle for the rapid and specific release of Ca^2+^, known as the Ca^2+^ reservoir (112). Accumulating evidence suggests that Ca^2+^ release from the ER is critical for mitochondrial dysfunction and increased mtROS (113, 114). Ca^2+^ transport between ER and mitochondria is mainly co-regulated by ER-mitochondria encounter structure (ERMES), Mfn2, inositol-1,4,5-triphosphate receptor (IP3R)/Grp75/voltage-dependent anion channel (VDAC), and MCU channels (115). ER release of Ca^2+^ is mainly mediated by IP3R, and then VDAC (located in the mitochondria outer membrane) is responsible for translocation from the mitochondrial outer membrane to the MCU. It was reported that histamine-stimulated IP3R mediates Ca^2+^ release from the endoplasmic reticulum to mitochondria, and the increased mitochondrial calcium concentration can enhance oxidative phosphorylation by activating four mitochondrial dehydrogenases, improving the metabolic efficiency of VSMCs, and modulate the phenotypic transition of VSMCs (116), and PKA/Mfn2 can regulate the connection between mitochondria and ER, the overexpression of Mfn2 can increase the functional coupling between the two organelles, which is conducive to the rapid flow of mitochondrial Ca^2+^ (116). Xia et al. found that nanosized hydroxyapatite (nano-HAp) can increase mitochondrial Ca^2+^ uptake in VSMCs, which in turn leads to excessive ROS production and oxidation of mtDNA (ox-mtDNA), and the release of more ox-mtDNA from mitochondria to the cytoplasm activates NLRP3/caspase-1/gasdermin D and induce VC by secreting inflammatory factors, antioxidants can effectively reverse this process and alleviate the progress of VC (33). In addition, the anticoagulants sodium citrate and diethyl citrate can also effectively reduce cell necrosis and lysosomal damage from [Ca2+]i overload induced by nano-HAp, avoiding further VC from cell death (29).

### Metabolic reprogramming

Metabolic reprogramming, considered to alter the activity of selected energy-producing or biosynthetic pathways, is a key regulator of VSMCs phenotypic transition and signal transduction processes (117, 118). Unusually, even though OXPHOS is much more efficient than glycolysis to generate ATP, a substantial proportion of ATP in VSMCs still originates from glycolysis rather than OXPHOS (118). The metabolism of VSMCs is characterized by a significant production of lactate even under fully oxygenated conditions. About 30% of the ATP supply in VSMC is derived from aerobic glycolysis, and 80–90% of the glycolytic flux contributes to lactate production (118, 119). VSMCs exhibit higher glucose uptake and aerobic glycolytic capacity during the phenotypic transition to maximize the energy supply required by cells (9, 120, 121). This increase in aerobic glycolysis resembles the Warburg effect in tumor cells, is an important feature of the transformation of multiple cellular phenotypes (122), and provides a potential target for clinical intervention. Although the recent view is contrary to Warburg’s original contention that increased aerobic glycolysis is not a consequence of ATP compensation of dysfunctional or damaged mitochondria (123). However, mitochondria’s healthy and larger presence enables them to compete with the LDH reaction for the intermediates of glycolysis (pyruvate and NADH), thereby minimizing lactate accumulation (124).

**FIGURE 3:**
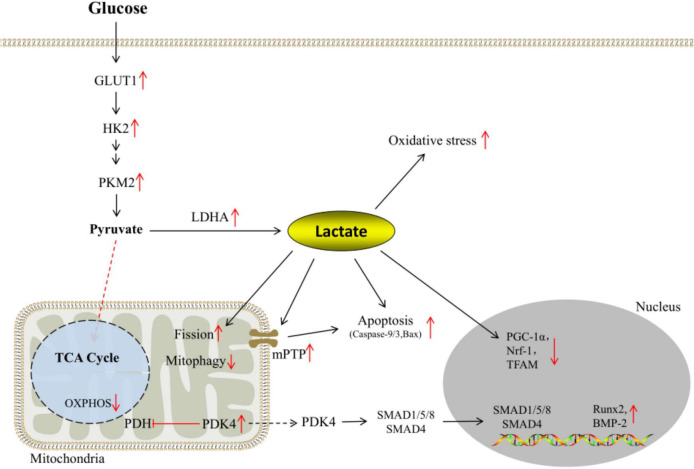
Reprogramming of glucose metabolism under calcifying conditions.

The expressions of GLUT-1 and glycolytic enzymes HK2 and PKM2 are up-regulated under calcifying conditions. Under the catalysis of LDHA, pyruvate is more inclined to generate lactate, the end product of glycolysis. Excessive lactate exposure not only leads to the imbalance of intracellular redox homeostasis and apoptosis of VSMCs, but also reduces the expression of biogenesis factors such as PGC-1α, Nrf1, and TFAM. Meanwhile, lactate impairs mitochondrial function through pathways such as mPTP opening rate and mitophagy. In addition, PDK4 is elevated under calcifying conditions, which inhibits OXPHOS. On the other hand, it promotes SMAD1/5/8 and SMAD4 to form heteromeric complexes and enter the nucleus, thereby regulating the transcription of osteogenic genes such as Runx2 and BMP-2 (Figure 3).

Glucose metabolism is critical for VSMCs phenotype regulation. Glucose uptake and lactate production increased during VSMCs calcification, suggesting an increased proportion of glycolysis in VSMCs glucose metabolism (9, 125). Multiple studies have demonstrated elevated blood lactate levels in diabetic patients (126, 127). Although we are currently unable to establish a direct link between blood lactate and diabetic VSMCs calcification, blood lactate concentrations can provide valuable insights into overall metabolic health (128). Mechanistically, lactate promotes Drp1-mediated mitochondrial fission and BNIP3-mediated mitophagy impairment by activating the nuclear receptor subfamily 4 group A member 1 (NR4A1)/DNA-dependent protein kinase, catalytic subunit (DNA-PKcs)/p53 pathway, ultimately leading to apoptosis and accelerate VSMCs calcification (11). In addition, lactate is closely linked to mitochondrial dysfunction and intracellular oxidative stress (68). Glucose uptake by cells is primarily through glucose transporters, followed by the conversion of glucose to pyruvate by the combined action of key rate-limiting enzymes such as hexokinase 2 (HK2) and pyruvate kinase in glycolysis. Pyruvate is then catalyzed by the pyruvate dehydrogenase complex for irreversible oxidative decarboxylation into acetyl-CoA, which enters the tricarboxylic acid cycle, or is catalyzed by lactate dehydrogenase to lactate. Glucose transporter1 (GLUT-1) is the major isoform in VSMCs. In β-GP-induced VC, the expression of GLUT-1 increased in a time-dependent manner and contributed to the proliferation of VSMCs (129, 130). Excessive GLUT-1 in humans results in a 44% increase in intracellular glucose concentration, similar to a diabetic environment (125, 131), while high intracellular glucose concentrations increase the expression of osteogenic transcription factors such as BMP-2, OPN, collagen type I, and ALP (125, 132, 133). In addition, the xxGLUT-1 polymorphism genotypes are associated with VC in early-stage kidney patients (125).

Hypoxia triggers VSMCs calcification and osteogenic differentiation in a HIF-1-dependent and mitochondrial ROS-dependent manner (134–136). As a key effector of hypoxia response, the hypoxia-inducible factor-1α (HIF-1α) can affect the transcription and activity of related metabolic enzymes and regulate the phenotypic transformation of VSMCs. In hypoxia, hydroxylation of HIF-1α is inhibited, and the accumulated HIF-1α binds to HIF-1 β to form a dimer, which in turn generates nuclear translocation and binds to specific DNA sequences of target genes (hypoxia response elements), and activate transcription of target genes (137). In human carotid smooth muscle, growth factors such as PDGF activate HIF-1 through PI3K/Akt/GSK3b pathway, hyperpolarized MMP by promoting HK2 translocation to mitochondria, inhibits OXPHOS of glucose, contributing to the increased of VSMCs glycolysis flux (138). In addition, activation of signal transducer and activator of transcription 3 (STAT3) could be a key event in the upregulation of HK2 activity and glycolysis in VSMCs, and inhibition of the STAT3/HK2 signaling axis can reduce glycolysis and inhibit VSMCs proliferation and migration (139). Pyruvate kinase isoform M2 (PKM2) is a crucial rate-limiting enzyme during glycolysis that accelerates VSMC proliferation and migration (140). In the Warburg effect, PKM2 not only inhibits mitochondrial Ca^2+^ levels, leading to the conversion of glucose metabolism from OXPHOS to aerobic glycolysis, but also promotes HIF-1α-mediated transcription of multiple metabolism-related genes such as lactate dehydrogenase (LDH)-A1 and GLUT-1 through nuclear translocation, ensuring that lactate levels are elevated (141). Pyruvate dehydrogenase kinase 4 (PDK4) is a mitochondrial matrix enzyme that negatively regulates the activity of the pyruvate dehydrogenase complex (PDC) through phosphorylation, thus contributing to mitochondrial function and glucose utilization in VSMCs (142). A growing number of studies have shown that the expression of PDK4 and aerobic glycolysis are increased in VSMCs calcification models induced by Pi or AGEs (143, 144). Recent evidences suggest that elevated PDK4 expression affects cellular metabolism and accelerates VSMCs calcification in at least the following ways: (1) PDK4 overexpression impairs mitochondrial function, manifested by decreased mitochondrial aerobic rate, ATP production, maximal respiration, and ROS-mediated apoptosis, resulting in decreased mitochondrial tricarboxylic acid cycle activity promotes the conversion of pyruvate to lactate production (30, 145); (2) PDK4 regulates the downstream signaling pathway of BMP2 and activate SMAD1/5/8 through PDK4 phosphorylation, resulting in phosphorylation SMADs translocating to the nucleus and promoting the transcription of osteogenic genes (146); (3) PDK4 increases in an AGEs/HIF-1α-dependent manner and promotes autophagy during VSMCs calcification (147), which may be a compensatory mechanism for cellular self-protection. PDK4 inhibition can decrease AGEs-induced ALP activity, Runx2 activation, and calcium deposition content and significantly downregulate lactate production (144, 148). Evidence suggests that interventions such as metformin and zinc supplementation can inhibit the osteogenic phenotype transition and calcification of VSMCs by reducing PDK4 expression levels (30, 149).

### Perspectives

In VSMCs, mitochondrial dysfunction can lead to oxidative stress, and metabolic transformation, triggering a series of cascade reactions. Therefore, targeting mitochondria is an attractive option in VC therapy. However, existing measures such as mitochondrial antioxidants have not yet shown satisfactory results in clinical trials. Zhang et al. believe that mitochondrial dysfunction is a dynamic network of changes, rather than damage to individual mitochondria (14). Meng et al. believe that the redox state of organelles is different and should be accurately detected, rather than non-specific treatment (150), how accurately assessing mitochondrial damage and regulating the balance of mitochondrial homeostasis is the key to future pharmacological research. In addition, lifestyle interventions such as exercise (14), fasting (151), and calorie restriction can improve mitochondrial function and balance redox homeostasis from the perspective of integrated physiology (152), which has broad application prospects.

Endoplasmic reticulum and mitochondria are physically and functionally closely linked, affecting the ROS generation, metabolism, and survival of cells *via* the regulation of ions such as Ca^2+^ and magnesium (Mg^2+^). Mg^2+^ is the second most abundant divalent cation in cells. As a natural antagonist of Ca^2+^, Mg^2+^ participates in the regulation of Ca^2+^ homeostasis competitively. In addition, Mg^2+^ acts as a cofactor for more than 300 metabolic responses and affects cardiovascular disease by regulating mitochondrial function, metabolism, oxidative stress, and inflammation (153). Daw et al. demonstrated that lactate acts as a signaling molecule to trigger dynamic changes in Mg^2+^ between the ER and mitochondria, thereby enhancing mitochondrial bioenergetics (154). Mg^2+^ supplementation has shown therapeutic potential in models of pulmonary arterial hypertension (155), but there is currently a lack of evidence in VC.

In conclusion, mitochondria play critical roles in cellular physiology, including metabolic flexibility, energy production, maintenance of redox homeostasis, calcium homeostasis processing, and cell death. Little is currently known about the interactions between mitochondria and other organelles and the mediating role of mitochondria between ions and metabolism, exploring the role of mitochondria in the phenotypic transition of VSMCs may provide clues for the precise prevention and treatment of VC.

## Author contributions

DW, YPL, and YZL conceived and designed the research. YZL, ZXL, and LLZ collected the manuscript data and designed the tables and images. YZL and ZXL wrote the manuscript. DW and YPL supervised the study and revised the manuscript. All authors contributed to the article and approved the submitted version.
